# Research activity, facilitators and barriers amongst trainee and early-career family physicians in sub-Saharan Africa: A cross-sectional survey

**DOI:** 10.4102/phcfm.v14i1.3367

**Published:** 2022-06-30

**Authors:** Pius O. Ameh, Chelsea M. McGuire, Alexandra van Waes, Bolatito B. Fatusin, Lara S. MacIntyre, Faith Lelei-Mailu, Hithaishini Kodicherla, Maame A. Egyirwa Buadu, Musa Dankyau, Kenneth Yakubu

**Affiliations:** 1 Department of Family Medicine, Federal Medical Centre, Keffi, Nigeria; 2 Department of Family Medicine, School of Medicine, Boston University, Boston, Massachusetts, United States of America; 3 Family Medicine Specialty Training Programme, School of Medicine, Lesotho-Boston Health Alliance, Leribe, Lesotho; 4 Department of Health Sciences, Boston University, Boston, Massachusetts, United States of America; 5 Department of Family Medicine, Faculty of Medicine, Federal Medical Centre, Gusau, Nigeria; 6 Department of Arts and Sciences, Tufts University, Boston, Massachusetts, United States of America; 7 Quality Health and Safety/Department of Family Medicine, Faculty of Medicine, AIC Kijabe Hospital, Kiambu County, Kijabe, Kenya; 8 School of Public Health, Boston University, Boston, Massachusetts, United States of America; 9 Department of Family Medicine, College of Medicine and Health Sciences, Bingham University and Bingham University Teaching Hospital, Jos, Nigeria; 10 Department of Family Medicine, Faculty of Medicine, University of Jos, Jos, Nigeria; 11 Department of Family Medicine, Faculty of Medicine, University of New South Wales, Sydney, Australia; 12 Department of Global Health, Faculty of Medicine, The George Institute for Global Health, Sydney, Australia

**Keywords:** research activity, research facilitators, research barriers, health research capacity strengthening, sub-Saharan Africa (SSA)

## Abstract

**Background:**

Primary health care systems in sub-Saharan Africa (SSA) need context-specific evidence to address current challenges. Increased family physician (FP) research activity could help fill this gap.

**Aim:**

To describe the research activity, facilitators and barriers amongst AfriWon Renaissance members.

**Setting:**

An online programme was designed to improve research activity amongst members of AfriWon Renaissance, an organisation of early-career and trainee FPs in SSA. This article provides a baseline description of their research activity.

**Methods:**

All AfriWon Renaissance members were invited to participate in an online survey. A content-validated study tool assessed research activity, including participation in research meetings, engagement in research mentorship, number of projects and published articles. Facilitators and barriers were assessed via Likert scales and two open-ended questions. The researchers conducted descriptive statistics using Epi Info 7, a content analysis of open-ended responses and triangulation.

**Results:**

Amongst the 77 respondents, 49 (63.6%) were still in training. Over two-thirds (71.4%) had participated in a research discussion in the past month. Whilst more than half (63.5%) reported having a manuscript under development, only 26 (33.8%) reported a recent publication. Nearly all (94.8%) intend to continue research in their FP careers. The most common facilitators were the institutional requirement to conduct research and having supportive peers and mentors. The most predominant barriers were time constraints and a lack of training on analysis.

**Conclusion:**

There is a cohort of committed young FP researchers who would benefit from efforts to address identified barriers and support for their ongoing research activity, in order to increase primary care research outputs in SSA.

## Introduction

There is a growing global recognition of the connection between strong health research capacity, translation of evidence into practice and the achievement of positive health care outcomes. Although health research has been linked to systems-strengthening and universal health care achievement, research capacity gaps remain pronounced in low-income and middle-income countries (LMICs), where health system strengthening is most needed.^1^ Within the African region, the World Organization of Family Doctors (WONCA) recognises that the regular conduct of research and clinical audits by Family Physicians (FPs) is key to strengthening primary care and district health services.^2^ However, despite the importance of research in primary care, there remains a relatively low research output in sub-Saharan Africa (SSA), hindering efforts to improve health services across the continent.^3^

Numerous health research capacity strengthening (HRCS) efforts have attempted to address this gap in research production in SSA, both in general and specific to primary care.^4,5,6,7^ However, the impact of such efforts on increasing research activity is often limited by a variety of factors. At the individual level, a lack of protected time for research, a high clinical workload and a lack of dedicated mentorship have been cited as reasons why FP trainees in SSA are unable to acquire research skills or pursue a career in research.^8,9,10^ At a systems level, HRCS efforts can be hindered by brain drain, a lack of local autonomy and ownership of a research agenda, high maintenance costs of research institutions and a dependence on foreign aid.^4^ A greater understanding of the specific factors affecting research and HRCS in SSA is important for developing context-relevant programmes that can increase capacity for primary health care research.^10^

In 2019, a pilot research training and mentorship initiative called AfriWon Research Collaborative Program was designed to increase the level of research activity amongst early-career FPs in SSA.^7^ Prior to commencing this pilot programme, the researchers conducted a survey to describe research activity, as well as facilitators and barriers to research within this population. This article describes the findings from this survey.

## Methods

### Study design

An online cross-sectional survey was conducted consisting of both closed and open-ended questions.

### Setting and study population

AfriWon Renaissance (AfriWon) is a professional organisation of early-career and future FPs under the aegis of a professional group of FPs in Africa. All survey participants consisted of AfriWon members who were either (1) trainees in a family medicine residency programme in SSA or (2) early-career FPs living in SSA who were within five years of completing their training. These survey responses were used as the baseline for a longitudinal evaluation of the AfriWon Research Collaborative Program, which will be further discussed in a forthcoming publication. As all modules and communication were in the English language, limited readers, nonreaders and nonspeakers of the English language were excluded.

### Recruitment

The researchers used a voluntary response sampling strategy which allowed participants to take part in the survey through a link that was distributed via the social media platforms of AfriWon and its email list. The survey was open for one month and participation was incentivised through a raffle draw for a primary care e-book.

### Tool development and data collection

The researchers generated survey items to describe research activity by adapting a previously published point system for family medicine trainee scholarly activity.^11^ Questions included the following:

- How many active research projects are you involved in right now?
- In this past month, have you participated in any research meetings, forums, chats or discussion groups?
- How many research protocols have you had approved by an institutional review board (IRB?)

Closed-ended questions on research mentorship and an item exploring plans for future research activity was also included. To describe facilitators and barriers to research, the researchers used common thematic areas from their review of the literature^9,12,13,14^ to design statements that participants then ranked on a 5-point Likert scale. Statements included the following:

- Research is undertaken by other health workers where I work.
- I have access to electronic databases.
- My employer or supervisor provides me with an adequate amount of time to conduct research.

The survey also included two open-ended questions asking for participants to state their three most important facilitators and barriers. See Online Appendix 1 for the entire survey tool.

Following the methods described by Zamanzadeh et al., the researchers calculated the content validity index of the study tool, retaining items with an item content validity index (I-CVI) score of greater than 0.79 and modifying those with I-CVI score of lesser than 0.79 for clarity.^15^ Data were collected using a Google Form linked to a password-protected Google spreadsheet. Data cleaning revealed only one duplicate participant, whose matched responses were consolidated and retained.

### Data analysis

#### Quantitative analysis

Descriptive analysis of demographic data was done using means, standard deviations, frequencies and proportions of data. The analysis was done using Microsoft^®^ Excel^®^ and Epi Info 7™.^16^ Missing values were few and were excluded via pairwise deletion.^17^ Participants’ research activity were displayed to view patterns and then dichotomised as a means of summarising the data.^18^ Likert scale data for facilitators and barriers were also dichotomised by recoding the ‘strongly agree’ and ‘agree’ items as ‘facilitator’ and the ‘neither agree or disagree’, ‘disagree’ and ‘strongly disagree’ as ‘barrier’. The researchers were interested in exploring if collected data suggested any differences in research activity or perceived facilitators and barriers between current trainees versus FP graduates. An exploratory bivariate analysis on dichotomised research activity and facilitator or barrier data was conducted to compare trainees and graduates in the sample.

#### Qualitative analysis

An inductive content analysis approach was used to analyse open-ended survey data on facilitators and barriers to research activity.^19^ Researchers L.v.W., H.K. and F.L-M. familiarised themselves with written responses and developed an initial codebook through independent open-coding of all data via constant comparison. They agreed on a finalised codebook and independently coded all data. After reaching consensus on codes, coded data and counts of the number of times mentioned by respondents were displayed as a grid of the major categories. These categories were then reviewed by the full research team to finalise the core findings. The online generator wordclouds.com was used to create a word cloud to visualise the most common as well as unique responses by inputting the verbatim or slightly modified open-ended survey responses.^20,21^ Reflexive memoing^22^ was used by L.v.W., H.K. and F.L-M. throughout the qualitative analysis process. This technique involves writing short notes of the researchers’ self-examination processes to tease out biases, predispositions and preferences.^22^

#### Triangulation of facilitators and barriers

The study compared and matched responses from the quantitative analysis of facilitators and barriers with the categories derived from the qualitative content analysis. The data were displayed in a table for interpretation in order to identify the facilitators and barriers that were most common across both analyses, as well as the novel ones identified through open-ended responses alone. The researchers used member checking^23^ to help ensure credibility of the analysis by emailing the triangulated research findings to all participants and incorporating their comments into the final analysis.

### Ethical considerations

Ethical clearance was obtained from the Boston University Institutional Review Board (Protocol H-38521) and the Federal Medical Centre, Keffi Health Research Ethics Committee in Nigeria (reference number FMC/KF/HREC/299/19). All respondents gave written informed consent prior to participation.

## Results

### Participant demographics

Amongst all participants (*n* = 77), the mean age was 37.9 years, 43 (55.8%) were female and 45 (58.4%) worked in an urban setting. The largest number of participants were from West Africa (52 participants, 67.5%). Trainees (*n* = 49) were younger, with a mean age 36.4 versus 43.3 amongst graduates (*n* = 28). The gender balance and regions of practice were similar between trainees and graduates. See Table 1 for collected demographics.

**TABLE 1:** Demographic characteristics of participants.

Demographic characteristic	All (*n* = 77)				Trainees (*n* = 49)				Graduates (*n* = 28)			
	Mean	s.d.	*n*	%	Mean	s.d.	*n*	%	Mean	s.d.	*n*	%
**Age** †	38.78	9.0	-	-	36.42	5.0	-	-	43.32	12.6	-	-
**Gender** ‡												
Male	-	-	33	42.9	-	-	21	42.9	-	-	12	44.4
Female	-	-	43	55.8	-	-	28	57.1	-	-	15	55.6
**Subregion of practice§**												
Southern Africa	-	-	10	13.0	-	-	7	14.3	-	-	3	11.6
East Africa	-	-	7	9.1	-	-	3	6.1	-	-	4	15.4
Central Africa	-	-	6	7.8	-	-	5	10.2	-	-	1	3.9
West Africa	-	-	51	66.2	-	-	34	69.4	-	-	17	65.4
High-income country¶	-	-	1	1.3	-	-	0	0.0	-	-	1	3.9
**Marital status§**												
Single	-	-	15	19.5	-	-	10	20.4	-	-	5	19.2
Married	-	-	53	68.8	-	-	33	67.4	-	-	20	76.9
Divorced	-	-	2	2.6	-	-	2	4.1	-	-	0	0.0
Separated	-	-	4	5.2	-	-	3	6.1	-	-	1	3.9
Other	-	-	1	1.3	-	-	1	2.0	-	-	0	0.0
**Primary place of employment§**												
Urban	-	-	45	58.4	-	-	30	62.5	-	-	15	55.6
Semi-rural	-	-	22	28.6	-	-	14	29.2	-	-	7	25.9
Rural	-	-	5	6.5	-	-	4	8.3	-	-	2	7.4
Other	-	-	3	3.9	-	-	0	0.0	-	-	3	11.1

Note: The table shows the demographic characteristics of participants in the study, with the first column showing all participants and the second and third columns showing the subgroups of our exploratory analysis: trainees and graduates. Standard deviations and percentages were calculated using Microsoft^®^ Excel^®^ and Epi Info 7™.

s.d., standard deviation.

¶ , One participant currently practises in a high-income country but is from the West African sub-region.

† , *n* = 73,

‡ , *n* = 76,

§ , *n* = 75 (*n* is less than 77).

### Research activity

Respondents in the study sample exhibited varying levels of research activity, as depicted in Table 2. Amongst all respondents, 50 (64.9%) had worked on one or more research projects in the preceding six months, with 47 (63.5%) having one or more manuscripts under development for publication. Fifty-five (71.4%) reported having participated in a research meeting or discussion in the past month. Approximately one-third, or 33.8%, had published one or more manuscripts in the preceding three years. Within the exploratory analysis, it was found that more graduates than trainees (53.6% vs 22.5%, *p* = 0.006) had published a manuscript; however, more trainees than graduates (88.0% vs 51.0%, *p* = 0.001) reported having a manuscript under development that they intended to publish. A similar, although less pronounced, trend in abstract submissions to conferences between these two groups were seen, with 17 out of 27 (63.0%) graduates having submitted at least one abstract compared to only 17 out of 47 (37.0%) of the trainees (*p* = 0.023). Responses to the likelihood of the respondent being involved in research in the future are also presented in Table 2. Respondents overwhelmingly indicated that they would likely be involved in research (94.8% in the full sample), with virtually no difference between subgroups.

**TABLE 2:** Reported research activity amongst participants.

Research activity item	All respondents		Trainees		Graduates		*p*
	*n*	%	*n*	%	*n*	%	
**Number of research projects worked on in the past 12 months**	-	-	-	-	-	-	0.258
0	27	35.1	19	38.8	8	28.6	-
≥ 1	50	65.0	30	61.2	20	71.4	-
**Number of manuscripts under development for publication in next 12 months†**	-	-	-	-	-	-	0.001
0	27	36.5	3	12.0	24	49.0	-
≥ 1	47	63.5	22	88.0	25	51.0	-
**Number of manuscripts published in the past 3 years**	-	-	-	-	-	-	0.006
0	51	66.2	38	77.6	13	46.4	-
≥ 1	26	33.8	11	22.5	15	53.6	-
**Number of research abstracts submitted to a conference, meeting or symposium in the past 3 years** ‡	-	-	-	-	-	-	0.023
0	41	54.7	30	63.8	10	37.0	-
≥ 1	34	45.3	17	36.2	17	63.0	-
**Have participated in a research meeting, forum or discussion group in past 1 month**	-	-	-	-	-	-	0.217
Yes	55	71.4	33	67.4	22	78.6	-
No	22	28.6	16	32.7	6	24.4	-
**Have a research mentor or mentors**	-	-	-	-	-	-	0.248
Yes	47	61.0	28	57.1	19	67.9	-
No	30	39.0	21	42.9	9	32.1	-
**Are a research mentor to another person§**	-	-	-	-	-	-	0.016
Yes	49	64.5	12	46.2	37	74.0	-
No	27	35.5	14	53.9	13	26.0	-
**How likely are you to participate in research as part of your family medicine career?¶**	-	-	-	-	-	-	0.538
Unlikely	4	5.2	3	6.1	1	3.6	-
Likely	73	94.8	46	93.9	27	96.4	-

Note: The table shows the results of a descriptive analysis of collected research activity variables amongst all respondents and an exploratory analysis of these variables within trainee and graduate subgroups. *P*-values for this exploratory analysis were calculated using Fisher’s exact test. The table also shows the reported likelihood of future participation in research amongst respondents.

¶ , Unlikely’ is the combination of ‘extremely unlikely’, ‘unlikely’ and ‘neither likely or unlikely’, whereas ‘likely’ is the combination of ‘extremely likely’ and ‘likely’.

† , *n* = 74,

‡ , *n* = 75,

§ , *n* = 76 (*n* is less than 77).

### Closed-ended facilitators and barriers to research activity

As seen in Table 3, having access to an IRB (63 of 77, or 81.8% positive responses) and electronic databases (59 of 77, or 76.6% positive response) were the two statements most frequently ranked as facilitators by respondents in the study sample. Most of the participants (63 of 77, or 81.8%) agreed that they felt confident performing a literature search. The least confidence was in conducting a qualitative data analysis, with only 29 of 77 (37.7%) agreeing that they were confident in doing this. Whilst slightly more than half of all respondents reported having access to research mentorship, 35 out of 77 people (45.5%) reported not having mentorship access. The statement most frequently reported as a barrier amongst all respondents was regarding protected time to conduct research, with 53 out of 76 (69.7%) respondents reporting their employers do not provide adequate time to conduct research.

**TABLE 3:** Research facilitators and barriers reported by participants.

Facilitator or barrier item†	All respondents		Trainees		Graduates		*p*
	*n*	%	*n*	%	*n*	%	
**I have access to research mentorship**							0.280
Disagree	35	45.5	24	49.0	11	39.3	
Agree	42	54.6	25	51.0	17	60.7	
**I have access to electronic databases I need to conduct a literature search**							0.137
Disagree	18	23.4	9	18.4	9	32.1	
Agree	59	76.6	40	81.6	19	67.9	
**I have access to software to help me collect, manage and/or analyse research data** ‡							0.472
Disagree	32	42.7	19	39.6	13	48.2	
Agree	43	57.3	29	60.4	14	51.9	
**My employer or supervisor provides me with an adequate amount of time to conduct research§**							0.509
Disagree	53	69.7	33	68.8	20	71.4	
Agree	23	33.3	15	31.3	8	28.6	
**I have access to a research ethics committee or institutional review board (IRB)**							0.605
Disagree	14	18.2	9	18.4	5	17.9	
Agree	63	81.8	40	81.6	23	82.1	
**I am confident in developing a research protocol**							0.248
Disagree	30	39.0	21	42.9	9	32.1	
Agree	47	61.0	28	57.1	19	67.9	
**I am confident in performing a literature search**							0.165
Disagree	14	18.2	11	22.5	3	10.7	
Agree	63	81.8	38	77.6	25	89.3	
**I am confident in conducting qualitative data analysis**							0.170
Disagree	48	62.3	33		15	53.6	
Agree	29	37.7	16		13	46.4	
**I am confident in conducting quantitative data analysis**							0.145
Disagree	35	45.5	25		10	35.7	
Agree	42	54.6	24		18	64.3	
**Research is undertaken by other health workers where I work§**							0.125
Disagree	20	26.3	10	20.8	10	35.7	
Agree	56	73.7	38	79.2	18	64.3	

Note: The table shows the number and proportion of respondents ranking each statement as a facilitator (agree) versus barrier (disagree). It shows the results of the exploratory analysis comparing these same facilitator and barrier rankings amongst trainees and graduates, with *p*-values calculated using Fisher’s exact test.

† , Disagree is the combination of ‘strongly disagree’ and ‘disagree’ or ‘neither agree nor disagree’, whereas ‘agree’ is the combination of ‘strongly agree’ and ‘agree’.

‡ , *n* = 75,

§ , *n* = 76 (*n* is less than 77).

This study exploratory analysis comparing reported research facilitators and barriers between trainees and graduates yielded no statistically significant differences. The biggest differences were seen in the degree to which other healthcare workers conduct research, with 38 out of 48 or 79.2% of trainees responding positively versus 18 out of 28 or 64.3% of graduates (*p* = 0.13). Increases in confidence were seen amongst graduates as compared to trainees across all steps of the research process. For example, whilst 28 out of 49 (57.1%) of trainees reported confidence in writing a research protocol, this increased to 19 out of 28 (67.9%) amongst graduates. Whilst confidence in quantitative analysis was primarily reported largely as a facilitator in both the full group and graduate group, it was reported largely as a barrier in the trainee subgroup (25 of 49 or 51.1% not confident).

### Open-ended facilitators and barriers to research activity

**FIGURE 1:**
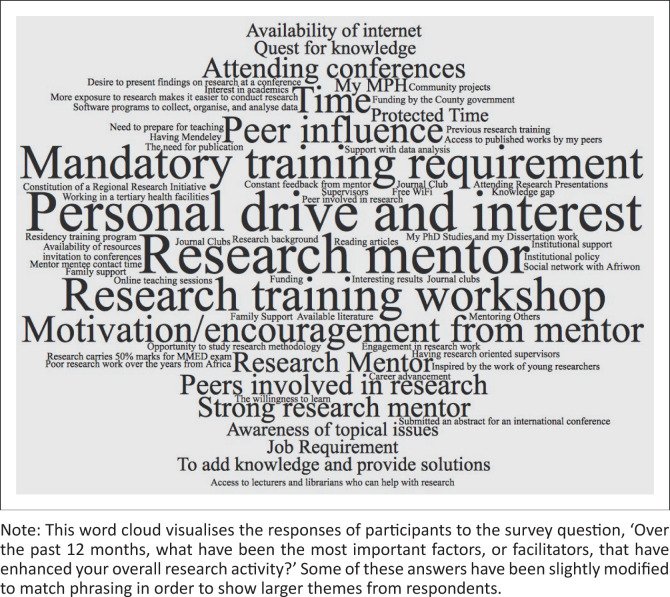
Word cloud of most important facilitators.

**FIGURE 2:**
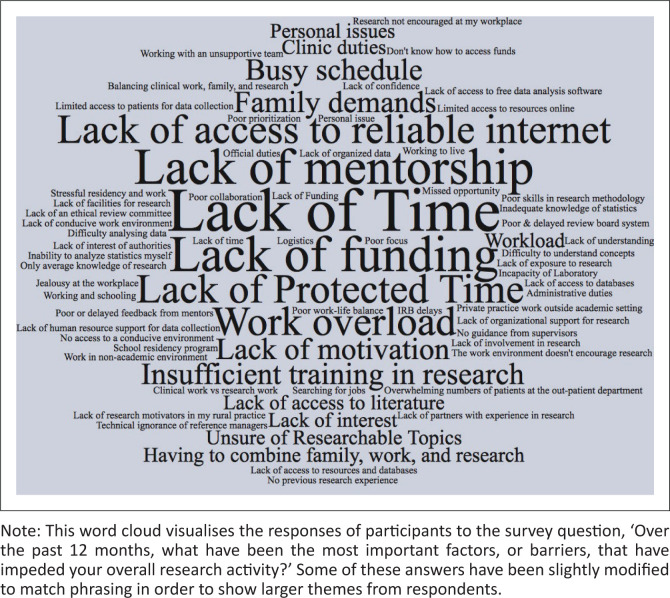
Word cloud of most important barriers.

From the analysis of the open-ended responses, the researchers derived eight categories of barriers and six categories of facilitators of research activity. Each category, the number of times it was mentioned and representative quotes are found in Online Appendix 2. The study highlights the most commonly mentioned categories, which can be visualised along with specific responses in Figures 1 and 2.

Facilitators reported by more than a quarter of respondents included the requirement to conduct research by one’s training programme or employer (23 respondents), positive influence of peers or other clinician-researchers (22 respondents), research mentorship (19 respondents) and the availability of research opportunities (19 respondents). The requirement to conduct research is exemplified by one participant here: ‘I’m a resident and I’m required to conduct an MMed research project’ (A1.43). Seventeen participants reported being facilitated by a personal drive to conduct research, such as this respondent: ‘The need to find solutions to problems faced in the course of practice’ (A1.35).

Two barriers were named by greater than a third of participants: (1) a lack of time, cited by 46 respondents, and (2) a lack of access to needed financial and other resources, cited by 32. The time constraint category included descriptions of a variety of competing demands on doctors’ time, as illustrated by one participant response:

‘Time. Teaching and attending to patients in the busy family medicine clinics as well as attending to family matters limit the time one has for research.’ (A1. 35)

Slightly fewer people (22) wrote about nonconducive research environments posing a barrier. The next most common barriers were a lack of research experience or training and a lack of proper mentors, both cited by 16. Some responses in the mentorship category acknowledge the interplay between a lack of time and mentorship:

‘The research mentor has other engagements like theatre sessions for emergency surgical procedures … so the research meeting with the mentor is usually cancelled.’ (A1.9-41)

### Facilitators and barriers triangulation

The facilitators that were most common across both the open-ended and Likert scale responses were: (1) access to adequate research mentorship and (2) working in an environment where peers conduct research or where it was required by the institution to conduct research. A lack of time was the most common barrier across both open-ended and closed-ended responses. This included a lack of protected time at the workplace, as was asked about in the Likert scale question. Respondents in the open-ended questions also spoke of time barriers from work overload and conflicting family or workplace responsibilities. Another common barrier was the lack of training on how to conduct research analysis, with specific reference to qualitative analysis in the closed-ended questions and a wider array of gaps in research and analysis skills noted in the open-ended answers. Table 4 highlights these triangulation results.

**TABLE 4:** Predominant facilitators and barriers determined via triangulation.

Facilitators	Barriers
Working in an environment that requires or encourages research	Lack of time (from work overload, conflicting responsibilities or a lack of protected time from the workplace)
Access to adequate research mentorship	Lack of training on how to conduct research analysis

Note: The table shows the predominant facilitators and barriers that were supported by triangulating findings from both the open-ended responses and the Likert scale questions.

The researchers noted an inconsistency between the open-ended and closed-ended responses with regard to the access to research resources. In the closed-ended questions, the participants largely agreed that they had access to online databases and software to manage and analyse data and had access to an IRB. However, in the open-ended responses, 31 participants noted a lack of access to finances and resources (including electronic databases, analysis software and research support personnel) as barriers to conducting research.

## Discussion

### Main findings

In this study it was found that a relatively high level of research activity, with over 70% having participated in at least one research meeting, forum, chat or discussion group in the preceding month. Additionally, all but four participants indicated they planned to be involved in research in the future. The majority of the activity was skewed towards the earlier stages of the research process (meetings, mentorship, manuscript preparation) with only about a third of the total sample reporting having published a manuscript. Approximately two-thirds of the sample, however, reported that they were working on a publication. The main facilitators of research activity were systemic or organisational, such as research being a requirement at one’s training institution or job and having adequate mentorship. The main barriers, such as time constraints and a lack of research analysis skills, could be considered individual-based factors or systemic factors, depending on the context.

### Overall research activity

The relatively high degree of early-stage research activity in this study sample of early-career African FPs was heartening, given the large amount of literature citing research capacity gaps amongst primary care physicians, particularly in LMICs, and in SSA.^1,9,10,24,25^ This finding may be largely explained by the target population. By sampling AfriWon members who were willing to volunteer for a survey on research activity, respondents are very likely to be biased towards those with an interest in research. In fact, research involvement is a major reason young FPs join the SSA professional physician group’s affiliate.^9^ It would be interesting to compare this finding with the research activity of non-AfriWon early-career FPs. A survey by Pawar et al. of research practices amongst 100 resident doctors from multiple specialties in India found that only 46% of respondents had protocol-writing experience and just 28% were involved in reading journals.^26^ Although not directly comparable research activity questions, this study finding that 88% of 49 trainees report having a manuscript under preparation likely suggests a greater degree of activity in this study population. The same survey found that 88% of resident respondents planned to carry out research in the future.^26^ A 2018 electronic survey of SSA surgical clinicians found similar results, with 92% reporting that they were likely to carry out future research.^27^ The similar findings reported in these two studies increase the validity of this study finding that approximately 95% of respondents plan ongoing research involvement.

### Early-stage activity

The finding that more respondents reported activity in the earlier stages of research, such as research meetings and mentorship, than they did later research dissemination activities, such as manuscript or abstract publications, also makes sense considering this study target population. Firstly, the majority of the sample were trainees who are at the beginning of both their clinical and research careers, so they may simply have not yet progressed to the publication phase yet. This is supported by the exploratory analysis which suggested that more graduates in this study sample had publications than did trainees. Amongst graduates in this study sample, however, only slightly more than half reported a research publication in the past three years. The same barriers to research activity identified via this study, such as time constraints and a lack of funding, are two of the common barriers to publication of research.^28^

Encouraging amongst this study findings is that approximately 64% of respondents had one or more manuscripts under development for publication. These findings suggest that there is a need for targeted efforts to support young physician-researchers from an early stage to be successful in transforming this intention to publish into actual publications. An example of this would be a new approach by the *African Journal of Primary Health Care and Family Medicine*, which seeks to increase the rate of successful publication by novice researchers by matching them with an editorial board member.^29^

### Research facilitators

The observation in this study that the main facilitators of research activity were environmental and social, such as research being a requirement at one’s training institution or job and having adequate mentorship, makes sense given the characteristics of the target population. The AfriWon population is mostly drawn from organised training and residency centres across SSA, which would therefore provide the structure in which participants’ research activity is taking place. Factors such as access to research mentorship, access to electronic databases and software, access to an IRB, confidence in developing a protocol and confidence in undertaking a literature search were all predominately ranked as facilitators on the Likert scale questions in varying proportions. Amongst these a complex interplay can be seen between external and environmental facilitators and personal facilitators.

Similar research facilitators were reported by other authors. Pawar et al. found that 66% of respondents were motivated by a ‘guiding senior faculty member’, who would no doubt be providing mentorship.^26^ Conradie et al.’s study found that ‘interactions with the research team’ were reported as a facilitator, which is similar to the facilitator of research networks and peer influence found in this study. One personal facilitator reported in the literature was ‘the personal desire to establish a research culture’.^27^ Whilst this study did not find this specifically, the researchers did identify a similar, albeit broader facilitator: the personal motivation to conduct research.

Whilst access to research mentorship in this study population was ranked as a ‘facilitator’ by a slim majority (approximately 55%), this leaves a considerable number of respondents reporting not having mentors. This is important because the research capacity literature is very clear that research mentorship is a crucial aspect of HRCS.^6,30^ To capitalise on these facilitators, the researchers recommend keeping the institutional structures that require academic research for FP training, whilst strengthening and expanding access to quality research mentorship.

### Research barriers

In this study, a lack of time was observed as a clear research barrier, whilst other factors, such as research analytical skills and access to resources, were more nuanced. The main barrier of time constraints because of work overload, conflicting responsibilities and/or a lack of protected time was unsurprising given that the researchers were drawing from health workers in SSA, who are often reported to be in short supply and overworked.^31^ This data suggests that early-career FPs would benefit from protected time in order to encourage research activity and outputs.

Confidence in conducting qualitative research was a barrier reported in the main analysis, as well as amongst both trainees and graduates in this exploratory analysis. This suggests the barrier may remain stable throughout the career of the FPs, surviving the transition from trainee to fellow. The lack of confidence in qualitative analysis may therefore correspond with a competency not gained during residency. This analysis suggested, however, that graduates were more confident in quantitative analysis skills than trainees. This may indicate there may be a qualitative analysis-specific gap in this study target population and an opportunity for targeted HRCS in this area.

Also, it was noted that the majority of participants reported that they did have access to specific research tools, such as literature databases or software to manage and analyse data, but in the open-ended response questions, a considerable number of participants reported that they did not have access to these because of financial or other reasons. It might be that the qualitative data collection method was able to retrieve more nuanced information here. Further study is warranted to better understand the specific research resources for which this population has the greatest need.

A lack of time and a lack of adequate resources or research facilities are well-supported barriers in the literature in similar populations.^26,27^ Other barriers in the literature, such as language barriers and difficulties with long-distance mentor–mentee relationships,^32^ were not found in this study, perhaps because the majority of AfriWon physicians communicate in English and maintain an online community.

### Study strengths

This study had a relatively large sample size of 77 participants, which compares well with literature.^26^ This, to the researchers knowledge, is the first SSA study that had representation from all regions, which would increase the generalisability of the findings. The robustness of the quantitative analysis was increased by using triangulation with open-ended responses.

### Study limitations

Recruiting the participants online could be limited by response bias, because the questionnaire might have only drawn the attention primarily of respondents who are interested in research. The study tried to minimise this bias by offering a raffle for a primary care e-book relevant to all participants’ practice context. This, it was presumed, would increase the number of respondents and incentivise those without a specific interest in the topic. In addition, the study was unable to calculate the response rate of the study because there was no updated database of all AfriWon members from which a denominator could be drawn. Of note, via the AfriWon social media platforms, approximately 300 AfriWon members were accessed, but were unable to reach the large number of early-career FPs in the subregion who might have otherwise met the study inclusion criteria. This number is significant; a particular African country alone has approximately 1500 FP trainees.^33^ This study exploratory analysis of trainees as compared to graduates resulted in smaller sample size for these two subgroups. This reduced the power of the analysis, and therefore these findings should be interpreted with caution. One notable limitation of the research activity tool was that it did not differentiate first-author publications from other-author publications; therefore, this study findings around authorship are nonspecific. Finally, the cross-sectional study method limits interpretation of findings in terms of causality.

### Future directions

More work is needed to identify the most promising strategies for addressing the specific barriers and capitalising on the facilitators identified. For example, whilst this study further confirmed that early-career FPs need more time for research, future work ought to explore the most effective way to provide this protected time. Similarly, further work could elucidate the best strategies for providing needed training on qualitative analysis to early-career FPs or how to better foster peer research networks. Another priority would be to develop a validated research activity tool that could be used for longitudinal evaluations of HRCS interventions designed to increase research activity in this population.

### Implications for policy and practice

More interventions are needed to support early-career FPs to overcome the barriers faced and capitalise on existing facilitators. Evaluations of HRCS interventions aimed at this population should aim to account for the early steps in the research process and not limit measurement to the conventional ‘outcomes’ which are often narrowly focused on number of publications.

## Conclusion

There is a population of young FP researchers who are committed to research careers and would benefit from targeted HRCS efforts to address the identified barriers of a lack of time and training. These efforts should build on enabling environments and expanding research mentorship in order to empower participants to reduce health challenges of SSA through primary care health research.
